# Efficacy of Azithromycin Eyedrops for Individuals With Meibomian Gland Dysfunction–Associated Posterior Blepharitis

**DOI:** 10.1097/ICL.0000000000000729

**Published:** 2020-07-07

**Authors:** Reiko Arita, Shima Fukuoka

**Affiliations:** Itoh Clinic (R.A.), Saitama, Japan; Lid and Meibomian Gland Working Group (R.A., S.F.), Tokyo, Japan; and Omiya Hamada Eye Clinic (S.F.), Saitama, Japan.

**Keywords:** Azithromycin, Meibomian gland, Meibomian gland dysfunction, Posterior blepharitis, Dry eye

## Abstract

**Purpose:**

To examine the safety and efficacy of azithromycin eyedrops in Japanese individuals with meibomian gland dysfunction (MGD)-associated posterior blepharitis.

**Methods:**

Individuals with MGD-associated posterior blepharitis who visited the Itoh Clinic, Saitama, Japan, were randomly assigned to receive azithromycin (1%) eyedrops (AZM group, 16 eyes of 16 patients) or preservative-free artificial tears (control group, 20 eyes of 20 patients) for 2 weeks. All subjects also applied a warming eyelid compress twice per day. Subjective symptoms (Standardized Patient Evaluation of Eye Dryness [SPEED] score), lipid layer thickness (LLT) and interferometric pattern of the tear film, plugging and vascularity of the lid margin, noninvasive break-up time of the tear film (NIBUT) and fluorescein-based break-up time of the tear film (TBUT), corneal–conjunctival fluorescein staining score, tear meniscus height, meibum grade, meiboscore, tear osmolarity, and Schirmer test value were determined before and after treatment. Side effects of treatment were also recorded.

**Results:**

In the AZM group, SPEED score, LLT, interferometric pattern, plugging and vascularity of the lid margin, NIBUT, TBUT, meibum grade, and tear osmolarity were significantly improved after treatment compared with baseline. The SPEED score, interferometric pattern, plugging, vascularity, meibum grade, and tear osmolarity were also significantly improved after treatment in the AZM group compared with the control group. Common side effects in the AZM group were transient eye irritation and blurred vision.

**Conclusion:**

Azithromycin eyedrops improved eyelid inflammation, the quality and quantity of the lipid layer of the tear film, and tear film stability. Such eyedrops thus seem to be a safe and effective treatment for MGD-associated posterior blepharitis.

Meibomian gland dysfunction (MGD) is the most common cause of dry eye and has a prevalence that varies widely from 3.5% to 70% according to age, sex, and ethnicity.^1^ A population-based study (Hirado-Takushima study) performed on Takushima island in Japan found the prevalence of MGD to be 32.9%.^2^ Meibomian gland dysfunction is a chronic condition of the meibomian glands that is characterized by terminal duct obstruction or qualitative or quantitative changes in glandular secretion (meibum).^3^ In the obstructive form of MGD, hyperkeratinization of the ductal epithelium results in a reduced availability of meibum to coat the aqueous layer of the tear film.^4^ This meibum deficiency thus gives rise to increased tear evaporation, tear hyperosmolarity, and increased bacterial growth at the lid margin.^5^

Treatment options for the initial conservative management of MGD include the application of a warm compress, the practice of lid hygiene, meibum expression, and, in more severe cases, the administration of anti-inflammatory drugs.^6^ The efficacy of topical azithromycin for the treatment of MGD has been attributed to its anti-inflammatory and antibacterial properties, which may suppress MGD-associated posterior blepharitis and bacterial growth on the eyelid.^6^ In addition, recent evidence suggests that azithromycin might directly induce the differentiation of and lipid production by meibomian gland cells, thereby leading to the alleviation of symptoms.^7,8^

Azithromycin is a macrolide antibacterial agent with high efficacy for *Cutibacterium acnes*. Azithromycin ophthalmic solution (Azimycin ophthalmic solution 1%; Senju Pharmaceutical, Osaka, Japan) possesses not only antibacterial but also anti-inflammatory and lipid-regulating effects,^9^ and it becomes widely distributed throughout and is readily retained by the eyelid.^10^ Although many studies have examined the efficacy of topical azithromycin for the treatment of posterior blepharitis,^9,11–19^ the possible effects of such treatment on multiple parameters such as the quality and quantity of the lipid layer of the tear film, tear film stability, lid margin abnormalities, meibomian gland morphology, tear osmolarity, and ocular symptoms, as compared either between before and after azithromycin instillation or between azithromycin-treated and control subjects, have not been determined. The purpose of this study was therefore to evaluate comprehensively the safety and efficacy of 1% azithromycin eyedrops in Japanese individuals with MGD-associated posterior blepharitis.

## PATIENTS AND METHODS

This prospective randomized study was conducted at Itoh Clinic in Saitama, Japan, adhered to the tenets of the Declaration of Helsinki, and was approved by the Institutional Review Board of the Faculty of Medicine at Itoh Clinic. The trial has been registered with the University Hospital Medical Information Network database (UMIN000037715). Written informed consent was obtained from all participants.

### Patients

Meibomian gland dysfunction–associated posterior blepharitis was defined on the basis of MGD diagnostic criteria in Japan^20^ as (1) the presence of any chronic ocular symptom, (2) the presence of vascularity of the lid margin, and (3) the obstruction of meibomian glands as revealed by the detection of plugging and reduced meibum expression in response to moderate digital pressure^21^ in at least one eye. Exclusion criteria included a known allergy to azithromycin, ocular infection, pregnancy, and ocular inflammation other than posterior blepharitis including conjunctivitis and keratitis. Only one eye of each subject was enrolled in the study; if both eyes were eligible, the left eye was selected. Participants who were qualified for enrollment on the basis of the inclusion and exclusion criteria underwent a wash-out period of 2 weeks, during which they were instructed to administer preservative-free artificial tears (Soft SanTear; Santen, Osaka, Japan) six times a day.

Participants were randomized according to a block-of-two randomization scheme into azithromycin and control groups. Randomization was concealed with the use of closed envelopes, which were opened after the wash-out period for each participant. In the azithromycin group, individuals received treatment with 1% azithromycin eyedrops (Azimycin) twice per day (in the morning and evening) for 2 days and then once daily (in the evening) for 12 days. Control subjects were instructed to administer preservative-free artificial tears (Soft SanTear; Santen, Osaka, Japan) four times a day. Both groups were instructed to apply a warm compress to the study eye twice per day. Preservative-free artificial tears and a warm compress have been recognized as effective for the treatment of MGD.^6^ To avoid potential bias, we explained to the study subjects that each treatment was effective for MGD. All participants were evaluated for ocular symptoms and MGD-related parameters both at baseline and after the 2-week treatment period as described below. The study treatment was initiated immediately after the baseline evaluation. The subjects were asked to apply the eyedrops and warm compress up to and including the night before the 2-week visit, but not to administer them on the day of the visit.

### Examinations

Ocular symptoms were assessed with the Standardized Patient Evaluation of Eye Dryness (SPEED) questionnaire.^22^ The thickness of the lipid layer of the tear film (LLT) was measured with the use of the LipiView interferometer (Johnson & Johnson, Stamford, CT). Lipid layer grade (0, normal type; 1, aqueous-deficient type; and 2, evaporative type)^23^ and noninvasive break-up time of the tear film (NIBUT) were determined with the DR-1α interferometer (Kowa, Aichi, Japan).^23^ Lid margin abnormalities,^24^ including plugging (scale of 0–3) and vascularity (scale of 0–3),^24^ were observed by slitlamp microscopy. Vascularity of the lid margin was graded according to the combination of the presence of redness in the lid margin conjunctiva and the distribution of telangiectasia crossing meibomian gland orifices, as we previously reported.^24^ The fluorescein-based break-up time of the tear film (TBUT), corneal–conjunctival fluorescein staining (fluo) score (scale of 0–9),^25^ tear meniscus height (TMH: scale of 0–2, corresponding to low, medium, or high, respectively), and grade of meibum expressed with digital pressure (scale of 0–3)^21^ were evaluated by slitlamp microscopy. The meiboscore (0–3 for each eyelid), which reflects the disruption of meibomian gland morphology, was determined with a noncontact meibography system (Topcon, Tokyo, Japan),^26^ the volume of tear fluid was measured using the Schirmer test performed without the administration of anesthetic,^27^ and tear osmolarity was measured at the palpebral conjunctiva with an I-PEN Osmolarity System (I-MED Pharma, QC, Canada).^28^ All participants in the azithromycin group were evaluated for drug side effects on the basis of a diary in which they were asked to record such potential adverse events during the treatment period.

### Statistical Analysis

Sample size was calculated on the basis of a mean difference in vascularity of 1.6 (with a corresponding SD value of 0.64) between the control group and the azithromycin group after treatment for 2 weeks, as well as of a mean change in vascularity of 1.8 (with a corresponding SD value of 0.55) for the azithromycin group between before and 2 weeks after treatment initiation. Vascularity was graded according to the presence of redness and range of distribution in each eyelid.^24^ These values were based on the findings of a pilot study with five eyes of five subjects in each group. On the basis of these assumptions, a sample size of 10 eyes per group would yield a power of greater than 90% to show a significant difference at the level of α=0.05 with a two-sample *t* test.

Data are presented as mean±SD. After confirmation of a nonnormal distribution of data with the Shapiro–Wilk test (*P*<0.05), nonparametric testing was performed. The Mann–Whitney *U* test was applied to compare numerical parameters between the control and azithromycin groups either at baseline or after the 2-week treatment period. The Wilcoxon signed-rank test was applied to compare numerical parameters between before and after intervention. The sex ratio at baseline and the change in interferometric class between before and after intervention for the control and azithromycin groups were compared with the Fisher exact test. (Eyes of interferometric class 1 or 2 at baseline that were assigned to class 0 after intervention were considered to have undergone an improvement in condition.) The chi-square test was applied to compare the distribution of TMH or interferometric class between the two groups. The primary endpoint of the study was vascularity. All statistical analyses were performed with JMP Pro version 14 software (SAS, Cary, NC). All statistical tests were two-sided, and a *P* value of less than 0.05 was considered statistically significant.

## RESULTS

A total of 36 participants with bilateral MGD were enrolled in the trial, none of whom was lost to follow-up or discontinued the study treatment. All the subjects were treated for both eyes and completed the final visit of the study protocol. Baseline characteristics of the study participants are shown in Table 1. There was no significant difference in any baseline characteristic between the azithromycin and control groups.

**TABLE 1.** Baseline Characteristics for the Azithromycin (AZM) and Control Groups of Study Subjects With Meibomian Gland Dysfunction–Associated Posterior Blepharitis

Characteristic	Control Group	AZM Group	*P*
	(n=20)	(n=16)	
Sex (male:female)	8:12	8:8	0.74
Age (years)	61.9±12.2	60.1±17.9	0.89
SPEED score (0–28)	11.5±2.5	12.8±3.8	0.31
Lipid layer thickness (nm)	67.6±24.4	59.4±26.3	0.34
NIBUT (s)	4.6±3.1	3.8±2.5	0.44
Interferometric pattern			
Class 0	5 (25%)	3 (19%)	0.71
Class 1	5 (25%)	6 (38%)	
Class 2	10 (50%)	7 (44%)	
Lid plugging (0–3)	1.7±0.7	1.4±0.7	0.32
Lid vascularity (0–3)	2.1±0.6	1.9±0.9	0.56
Tear osmolarity (mOsm/L)	320.7±25.4	331.1±17.4	0.21
Tear meniscus height			
Low	8 (40%)	8 (50%)	0.77
Medium	4 (20%)	2 (13%)	
High	8 (40%)	6 (38%)	
TBUT (s)	4.7±1.8	3.8±2.5	0.23
Fluo score (0–9)	0.8±1.0	1.4±1.5	0.31
Meiboscore (0–6)	4.2±1.5	3.2±1.8	0.10
Meibum grade (0–3)	1.8±1.0	1.4±0.7	0.15
Schirmer test value (mm)	12.4±11.7	6.5±7.6	0.22

Data are presented as mean±SD. *P* values were determined with the Mann–Whitney *U* test, the Fisher exact test, or the chi-square test.

fluo score, corneal–conjunctival fluorescein staining score; NIBUT, noninvasive break-up time of the tear film; SPEED, Standardized Patient Evaluation of Eye Dryness; TBUT, fluorescein-based break-up time of the tear film.

Vascularity of the lid margin, tear osmolarity, NIBUT, TBUT, and meibum grade were significantly improved after treatment compared with baseline alone in the azithromycin group (Table 2). Among these parameters, vascularity, tear osmolarity, and meibum grade were also significantly improved in the azithromycin group compared with the control group after treatment (Table 2). Both groups showed significant improvements in ocular symptoms (SPEED score), plugging of the lid margin, and LLT after treatment compared with before treatment (Table 2). Among these parameters, the SPEED score and plugging were also significantly improved in the azithromycin group compared with the control group after treatment. Lipid layer grade (interferometric pattern) measured with the DR-1α instrument was significantly improved in the azithromycin group compared with the control group after treatment (Table 3). The distribution of TMH determined semiquantitively with a slitlamp microscope did not differ between the two groups before or after treatment (Table 3). The proportion of subjects showing an improvement in lipid layer grade between before and after treatment was also significantly greater in the azithromycin group than in the control group (Table 4). The fluo score, Schirmer test value, and meiboscore did not differ between the two groups either before or after treatment, or in either group between before and after treatment (Table 2).

**TABLE 2.** Characteristics of the Azithromycin (AZM) and Control Groups Before and After Treatment for 2 Weeks

Characteristic	Group	Baseline		After Treatment			
		Mean±SD	*P* for AZM vs. Control	Mean±SD	Mean Change±SE	*P* vs. Baseline	*P* for AZM vs. Control
SPEED score (0–28)	AZM	12.8±3.8	0.31	5.8±3.0	−7.0±1.1	<0.001^b^	0.018^a^
	Control	11.5±2.5		8.1±2.5	−3.4±0.3	<0.001^b^	
LLT (nm)	AZM	59.4±26.3	0.34	71.0±23.5	21.3±2.6	0.028^a^	0.36
	Control	67.6±24.4		63.5±26.9	1.8±1.9	0.005^a^	
NIBUT (s)	AZM	3.8±2.5	0.44	7.2±3.3	4.1±0.3	<0.001^b^	0.16
	Control	4.6±3.1		5.7±3.0	0.9±0.2	0.081	
Lid plugging (0–3)	AZM	1.4±0.7	0.32	0.2±0.4	−1.7±0.1	<0.001^b^	<0.001^b^
	Control	1.7±0.7		1.0±0.6	−0.5±0.1	<0.001^b^	
Lid vascularity (0–3)	AZM	1.9±0.9	0.56	0.4±0.5	−1.3±0.1	<0.001^b^	<0.001^b^
	Control	2.1±0.6		2.1±0.7	0.0±0.0	1.0	
Tear osmolarity (mOsm/L)	AZM	331.1±17.4	0.21	309.1±18.8	−0.1±0.0	<0.001^b^	0.014^a^
	Control	320.7±25.4		326.4±15.4	0.0±0.0	0.76	
TBUT (s)	AZM	3.8±2.5	0.23	6.0±2.8	3.3±0.4	0.001^a^	0.19
	Control	4.7±1.8		4.8±2.2	0.9±0.2	0.81	
Fluo score (0–9)	AZM	1.4±1.5	0.31	0.8±1.4	−1.0±0.2	0.13	0.63
	Control	0.8±1.0		0.7±1.0	0.0±0.1	1.0	
Meiboscore (0–6)	AZM	3.2±1.8	0.10	3.1±1.8	−0.3±0.1	1.0	0.10
	Control	4.2±1.5		4.1±1.4	0.0±0.0	0.50	
Meibum grade (0–3)	AZM	1.4±0.7	0.15	0.4±0.5	−1.9±0.1	<0.001^b^	<0.001^b^
	Control	1.8±1.0		1.6±1.0	−0.6±0.1	0.16	
Schirmer test value (mm)	AZM	6.5±7.6	0.22	6.3±6.4	0.3±0.8	0.99	0.30
	Control	12.4±11.7		10.6±10.3	1.4±0.5	0.076	

*P* values were determined with the Mann–Whitney *U* test or Wilcoxon signed-rank test.

a *P*<0.05.

b *P*<0.001.

fluo score, corneal–conjunctival fluorescein staining score; LLT, lipid layer thickness; NIBUT, noninvasive break-up time of the tear film; SPEED, Standardized Patient Evaluation of Eye Dryness; TBUT, fluorescein-based break-up time of the tear film.

**TABLE 3.** Interferometric Pattern of the Tear Film and Tear Meniscus Height (TMH) for the Azithromycin (AZM) and Control Groups at Baseline and After Treatment

Characteristic	Baseline			After Treatment		
	AZM	Control	*P*	AZM	Control	*P*
Interferometric pattern						
Class 0	3 (19%)	5 (25%)	0.71	13 (81%)	7 (35%)	0.017^a^
Class 1	6 (38%)	5 (25%)		2 (13%)	5 (25%)	
Class 2	7 (44%)	10 (50%)		1 (6%)	8 (40%)	
TMH						
Low	8 (50%)	8 (40%)	0.77	8 (50%)	6 (30%)	0.18
Medium	2 (13%)	4 (20%)		6 (38%)	6 (30%)	
High	6 (38%)	8 (40%)		2 (13%)	8 (40%)	

*P* values were determined with the chi-square test.

a *P*<0.05.

**TABLE 4.** Change in the Interferometric Pattern of the Tear Film for the Azithromycin (AZM) and Control Groups Between Before and After Treatment

	AZM	Control	*P*
Interferometric pattern			
Effective	10 (77%)	2 (13%)	0.002^a^
Ineffective	3 (23%)	13 (87%)	

Eyes of interferometric class 2 (evaporative dry eye type) or class 1 (aqueous-deficient dry eye type) at baseline were categorized as showing an improvement (i.e., treatment was effective) if the interferometric pattern changed to class 0 (normal type).

The *P* value was determined with the Fisher exact test.

a *P*<0.05.

Drug side effects were reported in 12 of the 16 patients (75%) in the azithromycin group. The most common side effects were eye irritation (12 of the 16 patients, 75%) and blurred vision (8 of the 16 patients, 50%), which were reported during the first 2 days of treatment but had subsided by the third day, and they therefore did not affect the SPEED score. Two of the 16 patients (12.5%) in the azithromycin group also complained of constipation (Table 5).

**TABLE 5.** Side Effects of Azithromycin Eyedrops (n=16)

Side Effect	*n*	%	Duration (Days)
Ocular (eyes)			
Eye irritation	12	75	First 2 days
Blurred vision	8	50	First 2 days
Nonocular (subjects)			
Constipation tendency	2	12.5	For 14 days

## DISCUSSION

As far as we are aware, our study is the first to evaluate the safety and efficacy of azithromycin eyedrops in individuals with MGD-associated posterior blepharitis in Japan. Such eyedrops were approved by the US Food and Drug Administration for the treatment of conjunctivitis in 2007, and they have been widely prescribed for conjunctivitis and posterior blepharitis not only in the United States but also in many countries in Europe, West and Southeast Asia, and Oceania. Although the prevalence of MGD appears to be higher in East Asia than in other parts of the world,^29^ azithromycin eyedrops were not launched for the treatment of conjunctivitis and blepharitis in this region until their approval in Japan in 2019. Moreover, our study is the first randomized controlled trial to evaluate multiple ocular surface–related parameters including those indicative of the quality and quantity of the lipid layer of the tear film, tear film stability, inflammation, the morphology and function of meibomian glands, and the quantity of tear fluid before and after treatment in both experimental and control groups.

Azithromycin eyedrops significantly improved subjective symptoms and all objective signs measured with the exception of the fluo score, the meiboscore, and the Schirmer test value compared with baseline. Of note, tear osmolarity and vascularity of the lid margin, both of which are indicators of ocular surface inflammation, were also significantly improved in the azithromycin group compared with the control group as well as between before and after treatment. In addition, meibum grade, which is the most common clinically determined parameter of meibomian gland function, was also significantly improved in the azithromycin group compared with the control group as well as between before and after treatment. Lipid layer grade and NIBUT determined with the DR-1α interferometer indicate the quality of the lipid layer of the tear film and the balance between the lipid and aqueous layers,^30^ whereas LLT measured with the LipiView instrument is an indicator of the quantity of the lipid layer.^31^ We found that lipid layer grade, NIBUT, and LLT were improved in the azithromycin group after treatment, indicating that both the quantity and quality of the lipid layer were improved. Together, our results suggest that azithromycin eyedrops were effective for the treatment of MGD-associated posterior blepharitis, and that their efficacy might be attributable, at least in part, to an anti-inflammatory action that results in an improvement in the condition of the lipid layer of the tear film and consequent increase in tear film stability.

Our present results are consistent with those of previous studies showing that topical azithromycin is a safe and effective treatment for the management of moderate to severe MGD.^13,32–34^ To date, 11 clinical studies performed in the United States, Italy, Turkey, Thailand, and Iran between 2008 and 2020 have shown that azithromycin eyedrops are effective for MGD treatment. The parameters examined by these studies included ocular symptoms, TBUT, ocular surface staining, meibum quality, lid margin abnormalities, and Schirmer test value.^11–13,15,17,19,32,34–37^ Our results correspond well with those of the studies showing that ocular symptoms,^11–13,15,17,19,36,37^ TBUT,^12,15,17,19,34,36,37^ meibum quality,^11,15,17,19,37^ and lid margin abnormalities^11,13,34–36^ were significantly improved after treatment with azithromycin eyedrops. Whereas we found that the fluo score was not significantly improved after azithromycin treatment, some previous studies also demonstrated a significant improvement in this parameter.^15,17,19,34–37^ All these previous studies that showed an improvement in ocular surface staining prescribed azithromycin eyedrops for 30 days, whereas the eyedrops were administered for only 14 days in our study as a result of medical insurance constraints. We also found that the morphology of meibomian glands visualized by noncontact meibography (meiboscore)^26^ was not affected by azithromycin treatment. Although noncontact meibography provides an indicator of the quality and quantity of meibum,^38^ the meiboscore is only a semiquantitative measure with a scale of 0 to 3,^26^ and a treatment period of 14 days may not be sufficient to detect a change in this parameter. Quantitative analysis may be necessary to evaluate any change in the morphology of meibomian glands induced by azithromycin.^39^

Meibomian gland dysfunction is a complex disorder that is associated with various pathways of inflammation that contribute to the operation of a vicious cycle.^4^ Obstruction of meibomian glands, a core mechanism of MGD, thus results in an increase in intraglandular pressure and acinar epithelial stress that give rise to the release of proinflammatory mediators such as chemokines and other cytokines and consequent inflammation. The resulting deficiency in the lipid layer of the tear film leads to an increase in the evaporation of tear fluid and tear hyperosmolarity, which further promote ocular surface inflammation.^4^ Factors released by commensal bacteria, such as lipases and toxins, may then alter the composition of meibum and result in the secretion of lipid species, such as free fatty acids, that are highly toxic and serve as proinflammatory mediators at the ocular surface. Azithromycin is a macrolide antibiotic that possesses anti-inflammatory and immunomodulatory properties that are thought to ameliorate eyelid and ocular surface inflammation.^11,18^ It thus blocks the activation of nuclear factor–κB and the associated production of proinflammatory chemokines and cytokines such as tumor necrosis factor–α, interleukin (IL)-1β, IL-6, and IL-8 in the eyelid margin and conjunctiva.^6,9,40^ It also has lipase-inhibitory activity and therefore attenuates the production of detrimental free fatty acids at the ocular surface.^6^ Furthermore, some commensal bacteria including *Staphylococcus aureus* and *Staphylococcus epidermidis* produce cholesterol or wax esterase that damages the tear film by breaking down the natural meibum lipids.^9^ Topical azithromycin shows bactericidal activity against such lid-associated bacteria. Moreover, it also stimulates the differentiation of meibomian gland epithelial cells as well as the accumulation of lipid in these cells and its eventual secretion.^41^

Although we found that azithromycin eyedrops were safe and effective in the study patients, there was a high rate of side effects such as eye irritation and blurred vision. However, most of these adverse effects were relatively minor and transient, and they did not result in discontinuation of the medication. In addition, as azithromycin is an antibiotic, there is a possibility of developing bacterial-resistant strains of bacteria with long-term use. However, it should not be a problem with only 2-week use.

Limitations of this study include the relatively small number of subjects, although the sample size calculated in advance was achieved. In addition, the follow-up period was short. Multicenter studies with a larger number of patients and with longer follow-up periods will be necessary to confirm our findings.

In conclusion, we found that azithromycin eyedrops significantly improved signs and symptoms of individuals with moderate to severe MGD. They thus ameliorated eyelid inflammation and increased the quality and quantity of the lipid layer of the tear film as well as tear film stability. Topical azithromycin thus seems to be a safe and effective treatment for MGD-associated posterior blepharitis.
